# Development and evaluation of an electronic nursing documentation system

**DOI:** 10.1186/s12912-021-00790-1

**Published:** 2022-01-10

**Authors:** Mohsen Shafiee, Mostafa Shanbehzadeh, Zeinab Nassari, Hadi Kazemi-Arpanahi

**Affiliations:** 1 Department of Nursing, Abadan University of Medical Sciences, Abadan, Iran; 2 Department of Health Information Technology, School of Paramedical, Ilam University of Medical Sciences, Ilam, Iran; 3 Department of Health Information Technology, Abadan University of Medical Sciences, Abadan, Iran; 4 Department of Student Research Committee, Abadan University of Medical Sciences, Abadan, Iran

**Keywords:** Nursing records, Nursing process, Electronic health record, Electronic medical records, Hospital information system

## Abstract

**Background:**

Nursing documentation is a critical aspect of the nursing care workflow. There is a varying degree in how detailed nursing reports are described in scientific literature and care practice, and no uniform structured documentation is provided. This study aimed to describe the process of designing and evaluating the content of an electronic clinical nursing documentation system (ECNDS) to provide consistent and unified reporting in this context.

**Methods:**

A four-step sequential methodological approach was utilized. The Minimum Data Set (MDS) development process consisted of two phases, as follows: First, a literature review was performed to attain an exhaustive overview of the relevant elements of nursing and map the available evidence underpinning the development of the MDS. Then, the data included from the literature review were analyzed using a two-round Delphi study with content validation by an expert panel. Afterward, the ECNDS was developed according to the finalized MDS, and eventually, its performance was evaluated by involving the end-users.

**Results:**

The proposed MDS was divided into administrative and clinical sections; including nursing assessment and the nursing diagnosis process. Then, a web-based system with modular and layered architecture was developed based on the derived MDS. Finally, to evaluate the developed system, a survey of 150 registered nurses (RNs) was conducted to identify the positive and negative impacts of the system.

**Conclusions:**

The developed system is suitable for the documentation of patient care in nursing care plans within a legal, ethical, and professional framework. However, nurses need further training in documenting patient care according to the nursing process, and in using the standard reporting templates to increase patient safety and improve documentation.

## Background

Clinical documentation and access to reliable information are crucial facets of nursing decision-making in care practice [1]. Nurses and other caregivers aim to exchange information about patients and administrative activities with high quality standards such as precision, timeliness, concurrency, conciseness, comprehensiveness, organization, and confidentiality [2]. Nursing documentation is defined as written evidence demonstrating that the nurse’s authorized and moral responsibilities were met in order for care to be assessed [3]. Accurate and comprehensive documentation of nursing interventions is essential for several other reasons. It improves patients’ outcomes, increases the quality and safety of healthcare services, ensures practice accountability, and facilitates communication between various involved health care stakeholders [4]. Accordingly, the nursing documentation framework needs to be standardized, reasonably organized, and structured to mirror the phases of the nursing process, i.e., the assessment, diagnosis, planning, implementation, and evaluation of patient conditions [5]. Despite the importance of clinical documentation, currently, there are undesirable situations of care recordings and a lack of an appropriate framework for documenting nursing care [6]. Studies in Iran have revealed that the nursing documentation is not compliant with the standards. Several studies compared international standards with those reported in Iranian studies showed that nurses in other countries were more diligent in following documentation principles and standards [7]. Currently, the clinical nursing reporting in most hospitals in Iran is paper-based which may be similar to writing a story with a varied and inconsistent format. In most cases, there is no legislative defense for the nursing staff because of the gradual fading and illegibility of manual records [8, 9]. Writing a nursing report is a routine event that that should be performed several times during a nurse’s daily work, therefore, nurses spend about 37% of their entire working time writing reports [10–12]. One-half of all nurses must stay at work for 1–2 h after the end of their shifts, mainly to complete nursing records [13]**.** This approach has several drawbacks including wasted time, disruption in patient care, medical errors, endangering patients’ safety, fading and illegibility of the paperwork, high staff turnover rates, legal problems, and, other similar factors [14, 15]. Due to the fast developments in information technology, the health industry actively attempts to employ electronic medical records (EMRs) for clinical practice, research, education, and supervision purposes. The Nursing information system (NIS), as a module of EMR, control nursing care or services and manages the nursing activities through which data are assembled, exchanged, stored, extracted, presented, and transferred [16]. It has been revealed that employing an EMR and electronic clinical nursing documentation leads to higher quality, more complete, and more patient-centric documentation than manual nursing documentation. National Health information technology officials suggested the use of standard terminologies and data sets to enable interoperability across health information systems. Several terminologies exist that support nursing practice, but none have been broadly leveraged in Iran’s E-health system [17]. To comply with data standards, nursing specialists must decide which data items should be included in the context of patient care, and what required data could be documented by other healthcare staff [18, 19]. Therefore, to overcome the limitations of above-mentioned paper-based nursing documentation, some considerations must be addressed from a data management standpoint [20, 21]. Thus, the purpose of this study was to design, develop, and evaluate of an electronic clinical nursing documentation System (ECNDS) and determine its core data elements and the validity of their corresponding values.

## Methods

### Study design

A four-step sequential methodological approach was utilized. The minimum data set (MDS) development process consisted of two phases, as follows: First, a literature review was performed to attain an exhaustive overview of the relevant elements of nursing and map the available evidence underpinning the development of MDS. Then, the data included from the literature review were analyzed using a two-round Delphi study with content validation by an expert panel. Afterward, a web-based system with modular and layered architecture was designed based on the derived MDS. Finally, to evaluate the developed system, a survey of 150 registered nurses (RNs) was conducted and the positive and negative impacts of the system were identified.

#### Literature review

**Fig. 1:**
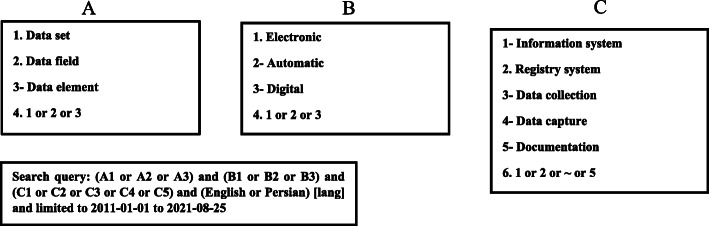
Search strategy

We performed a literature review to define the MDS-nursing report parameters. To conduct the study, first, an extensive literature review was performed in internet databases such as Web of Science, PubMed, ProQuest, Scopus, Magiran, and SID to identify the electronic nursing documentation system’s potential data elements. Thus, this step comprises all the elements associated with clinical nursing report templates and, it is necessary to incorporate and collect all elements related to nursing practices including diagnosis, assessment, and intervention. Inclusion criteria were (1) - availability of the full text of the journal articles, (2) - language (English or Persian), and (3) - publication date (from 2011 or later). Figure 1 shows the search strategy for identifying the relevant articles. The first part (Part A) included terms used for reporting templates. In the second part (Part B), the keywords related to digitalization were used. The third part (Part C) contained terms about studies on information system data architecture. The results of these three parts were combined using the Boolean operator “AND, OR”. The search was supplemented via checking the bibliographies of the identified articles.

Furthermore, previously developed relevant classification systems were systematically screened to collect information on clinical nursing report data elements, including the nursing diagnosis classification of the North American Nursing Diagnosis Association International (NANDA-I), and the Nursing Outcome Classification (NOC), Nursing Intervention Classification (NIC) and International Classification of Nursing Practice (ICNP). Finally, data fields were extracted from the related retrieved resources and entered into a checklist with two administrative and clinical sections.

#### Delphi phase

The initial MDS content was validated by the Delphi technique using two rounds as bellow:

### Participants

Using purposive sampling, we convened nursing professionals working as university faculty members of nursing in Iran’s Universities of Medical Sciences. Table 1 shows the demographic characteristics of these experts.

**Table 1:** Demographic characteristics of Delphi participants

Variables	frequency	percentage
gender		
female	43	53.75
male	37	46.25
Educational		
PhD nursing (RN)	74	92.5
Master nurse (RN)	6	7.5
Age		
30–40	19	23.75
40–50	48	60
> 50	13	16.25
Work experience in clinical field (years)		
< 10	12	15
10–15	28	35
15–20	24	30
20–25	8	10
> 25	8	10
Total	80	100

Demographic characteristics of Delphi participants

### Questionnaire development

The preliminary literature review provided the framework for developing a questionnaire to seek experts’ individual views regarding the important elements to be included in the MDS. The participants were asked to assess all items’ importance in the preliminary data list extracted from the literature review. Item importance was assessed based on a three-point Likert scale, which included three options: “yes”, “no”, and “unsure”. “yes” indicated the high importance of an element, and “no” meant the low importance of the element for inclusion. A blank row was provided at the end of the questionnaire for the experts to add the necessary data elements. The content validity of the questionnaire was assessed by an expert panel, including 80 nurses. Moreover, a test-retest was used to evaluate the reliability of the questionnaire. A consensus was reached based on experts’ agreement level regarding data elements to select items with ≥70% agreement (on an item’s importance) [22, 23].

### Delphi survey rounds

After the initial ranking, items with less than 60% agreement were deleted, those with more than 75% agreement were excluded from the second round, and those with 60 to 75% agreement were surveyed in the second round. The checklists were individually presented to the experts who were blind to the scores of the other experts, and if there was 75% consensus over a subject, it was included in the final MDS.

### Statistical analysis

Data were analyzed using the statistical package for social sciences (SPSS) software version 25 (Chicago, USA) via a few descriptive and analytical tests (chi-square, t-test, and paired t-test). The software was used to summarize respondents’ characteristics and demographic details. For each item outcome, the median, mean, and proportion ratings were calculated. To rank the scores, the median for each item outcome was calculated. The statistical significance was considered at *p* < 0.05.

#### Nursing documentation software development tools

Using Visual Studio 2019 a web-based nursing documentation system was designed. We used this platform because of its numerous benefits (e.g., cost-effective, scalable and accessible, user-friendly, fast and convenient, possessing the ability to custom search, improved Intellicode, having a clipboard, and refactoring attributes) [24]. The developed system was implemented with the cascading style sheets (CSS) technology as a web-based program. CSS, along with the hypertext markup language (HTML), was used to describe the presentation of documents and set the document syntax, layout, display format, and visual effects (e.g., font type, color, spacing, and sizes). The code was written in JavaScript language to design the website. Finally, Structured Query Language (SQL) was employed to develop the relational database (RDB). SQL provides efficient and systematic storage of data with high performance, availability, scalability, flexibility, management, and security [25].

#### Evaluation of the developed ECNDS

A pilot study was designed to assess clinical nurses’ views on working with the system. A total of 150 RNs participated in this survey, who worked in a variety of clinical wards, including; the emergency department, critical care wards, and other medical-surgical wards. The instrument used in this survey was a questionnaire containing 35 items which was employed to evaluate system quality, system usefulness, and user satisfaction of the ECNDS prototype system [26]. A five-point Likert scale was used to rate responses ranging from ‘never/almost never/not at all’, to ‘always/almost always/very great’ for each item. In the first section of the survey, respondents listed their credentials, primary work settings, job classification(s), and years of experience as a nurse.

The second section of the questionnaire contained 12 questions for assessing the ‘system usefulness ‘, which is defined as the frequency of using the system to complete patient care-related tasks. The third section of the questionnaire included 12 items for evaluating the ‘quality of the system, which is defined as the assessment of the quality of the system, its outputs, and its responsiveness. The last section of the survey had 11 questions on ‘user satisfaction with ENDS’, which is defined as the extent to which nurses believe the system is important in improving their work [26].

### Ethical considerations

The director of the research facility of the university approved the research protocol (approval ID: IR.ABADANUMS.REC.1400.065; date: 14/05/2021). All the participants were required to sign a confidentiality agreement and study participation consent form before joining the expert panel. They were cognizant of the objectives of the study. They were also informed that their participation was optional, and they had the liberty to withdraw from the study at any time.

## Results

### Phase 1: literature review

**Fig. 2:**
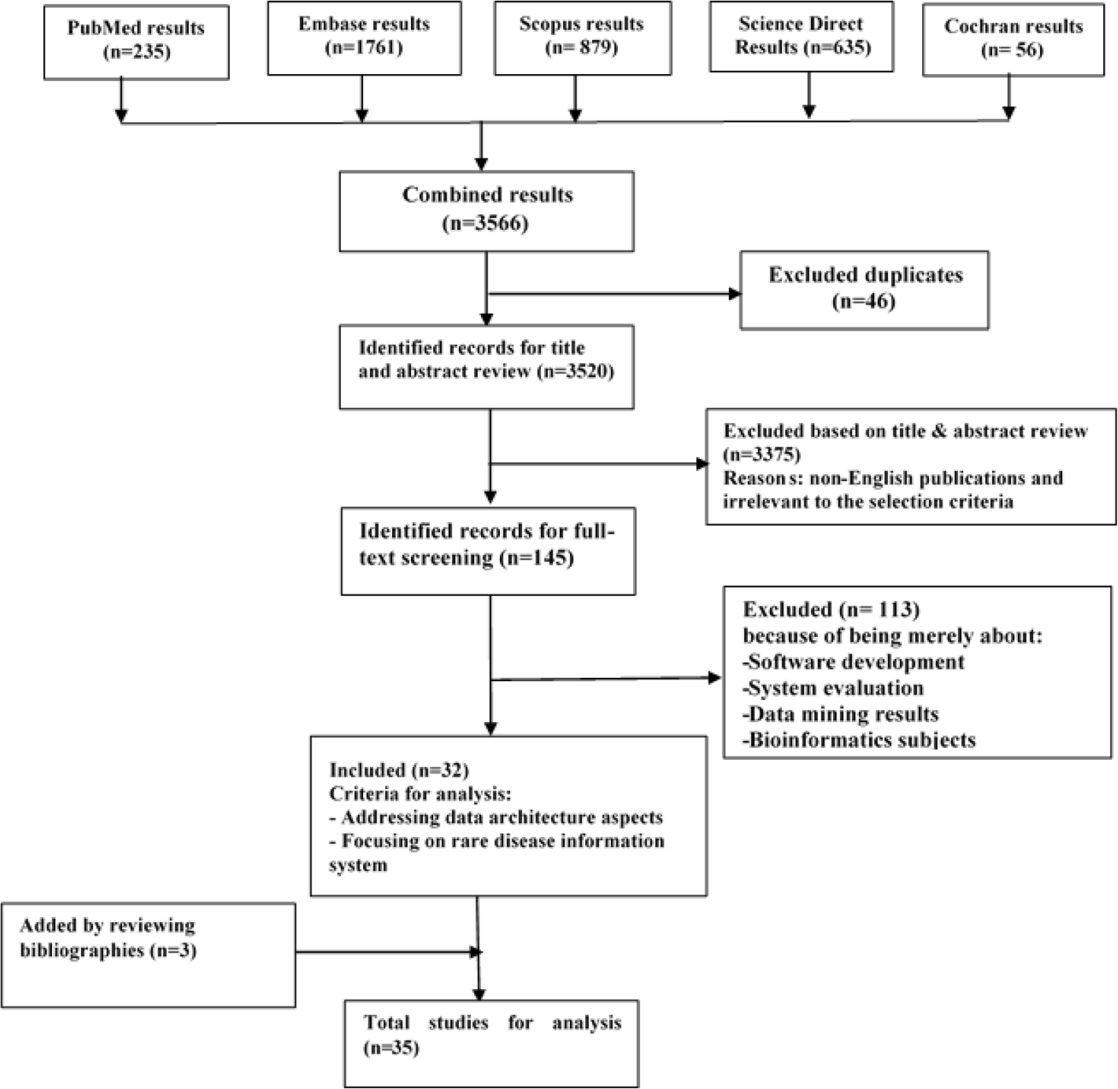
Search flow diagram

Searching the online databases resulted in retrieving 3520 articles from PubMed, Embase, Scopus, Science Direct, and Cochrane databases after removing the duplicates. Initial screening of the titles and abstracts resulted in 145 articles, of which 113 were excluded because they did not address reporting template items in relation to nursing practices. Three more articles were identified through checking the bibliographies, leading to a total of 35 articles for full-text review (Fig. 2).

### Phase 2: Delphi stage

In this study, after searching in scientific databases and according to the processes provided by NANDA, CCC, and ICNP nursing classifications, we extracted a set of data elements and validated them via a two-round Delphi survey for inclusion in the final MDS of nursing documentation. We divided this dataset into three general categories, including the administrative category, nursing assessment, and nursing diagnoses. The number of participants in the Delphi stage was 80 subjects, including 74 individuals with a nursing Ph.D. degree, and six nurses with an MSC nursing degree. About 43% of the participants were female, 88% of them had more than 10 years of clinical experience, and all participants had an RN degree (Table 1).

### Administrative information

This section has 19 items in our study. The items were delivered by the Delphi survey for nursing professionals. In the first round of Delphi, all the items of this section were confirmed (Table 2).

**Table 2:** Administrative and Nursing assessment information

Administrative information									
patient demographic information			Round 1			Round 2			Final Decision
			Agree N (%)	Dis agree N (%)	Unsure N (%)	Agree N (%)	Dis agree N (%)	Unsure N (%)	
Patient name, age, gender, educational level, Marital Status, Employment status, admitted date, admitted time, admitting nurse, Admitting physician			100	0	0				kept
Physician, Ward, Bed, Hospital name, Phone, Insurance, Source of history			100	0	0				kept
Past medical history			85.62%	8.4%	5.98%				kept
Items related to the nursing assessment									
Body systems	Item numbers (total)	Item numbers	Round 1			Round 2			Final Decision
			Agree N (%)	Dis agree N (%)	Unsure N (%)	Agree N (%)	Dis agree N (%)	Unsure N (%)	
Cardiovascular system	140	1–60	95.63%	2.4%	1.97%				kept
		61–100	89.87%	6%	4.13%				kept
		101–130	88.6%	10.7%	0.7%				kept
		131–140	69.43%	29.11%	1.46%	85.46%	14.54%	0	kept
Muscle and Skeletal system	80	1–40	98.32%	0	1.68%				kept
		41–68	92.87%	7.13%	0				kept
		69–78	90.56%	7.4%	2.04%				kept
		79–80	62%	36%	2%	65.36%	34.64%	0	remove
GU system	85	1–30	98.1%	0	1.9%				kept
		31–56	96.23%	0	3.77%				kept
		57–82	97%	0	3%				kept
		83–85	70.25%	29.75%	0	65.66%	34.34%	0	remove
Renal system	50	1–36	89%	10%	1%				kept
		37–45	90.39%	0	9.61%				kept
		46–50	98.85%	0	1.15%				kept
Neurological system	110	1–48	99%	0.5%	0.5%				kept
		49–88	91.41%	2.36%	6.23%				kept
		89–100	95%	4%	1%				kept
		101–108	88.64%	11.36%	0				kept
		109–110	67%	30%	3%	69.75%	30.25%	0	remove
Psychological and social	65	1–42	98.12%	1.88%	0				kept
		43–58	96.55%	2.32%	1.13%				Kept
		59–65	100%	0	0				Kept
Skin system	55	1–49	100%	0	0				kept
		50–55	96%	0	4%				kept
General appearance	150	1–36	100%	0	0				kept
		37–88	94.96%	5.04%	0				kept
		89–95	98%	2%	0				kept
		96–106	96%	3%	1%				kept
		107–120	86.76%	0	13.24%				kept
		121–134	79.23%	20.77%	0				kept
		135–145	89%	11%	0				kept
		146–150	88%	12%	0				kept
Respiratory system	140	1–55	89.51%	9.39%	1.1%				kept
		56–78	98.23%	1.77%	0				Kept
		79–100	96.37%	3.2%	0.43%				Kept
		101–125	95%	0	5%				Kept
		126–140	70.26%	21.36%	8.38%	79.80%	20.2%	0	kept
		35–40	89%	10%	1%				kept

Administrative and Nursing assessment information

### Nursing assessment information

This section has a total of 875 data items, which are divided into nine categories. These categories include (Table 2):

A. Cardiovascular system:
  The cardiovascular system had 140 data items. Items 1 to 60 (95.63% agreement), items 61 to 100 (89.87% agreement), items and 101 to 130 (88.6% agreement) were accepted in the first stage of Delphi; items 131 to 140 (85.46% agreement) are accepted in the second stage of Delphi, and finally, all 140 cardiovascular items were accepted. Each item had a percentage of acceptance, and the items whose acceptance percentage was close to each other are listed in a column.
B. Muscle and skeletal system
  The muscle and skeletal system had 80 data items. Items 1 to 40 (98.32% agreement), items 41 to 68 (92.87% agreement), and items 69 to 78 (90.56% agreement) were accepted in the first stage of Delphi. Items 79 to 80 (65.36% agreement) were removed in the second stage of Delphi and finally, 77 muscle and skeletal items were accepted. Each item had a percentage of acceptance that those items whose acceptance percentage was close to each and the listed in a column.
C. Genitourinary (GU) system
  The GU system had 85 data items. Items 1 to 30 (98.1% agreement), items 31 to 56 (96.23% agreement), and items 57 to 82 (97% agreement) were accepted in the first stage of Delphi. Items 83 to 85 (65.66% agreement) were removed in the second stage of Delphi and finally, 82 GU items were accepted. Each item had a percentage of acceptance and the items whose acceptance percentage was close to each other are listed in a column.
D. Renal system
  The renal system had 50 data items. Items 1 to 36 (89.1% agreement), items 37 to 45 (90.39% agreement), items 46 to 50 (98.85% agreement) were accepted in the first stage of Delphi, and therefore, all 50 renal items were accepted. Each item had a percentage of acceptance and the items whose acceptance percentage was close to each other are listed in a column.
E. Neurological system
  The neurological system had 110 data items. Items 1 to 48 (99% agreement), items 49 to 88 (91.41% agreement), items 89 to 100 (95% agreement), and items 101 to 108 (88.64% agreement) were accepted in the first stage of Delphi. Items 109 to 110 (69.75% agreement) were removed in the second stage of Delphi and finally, 108 neurological items were accepted. Each item had a percentage of acceptance and the items whose acceptance percentage was close to each other are listed in a column.
F. Psychological and social category
  The psychological and social category had 65 data items. Items 1 to 42 (98.12% agreement), items 43 to 58 (96.55% agreement), and items 59 to 65 (100% agreement) were accepted in the first stage of Delphi, and therefore, all 65 psychological and social items were accepted. Each item had a percentage of acceptance and the items whose acceptance percentage was close to each other are listed in a column.
G. Skin system
  The skin system had 55 data items. Items 1 to 49 (100% agreement) and items 50 to 55 (96% agreement were accepted in the first stage of Delphi, and therefore, all 55 skin items were accepted. Each item had a percentage of acceptance and the items whose acceptance percentage was close to each other are listed in a column.
H. General appearance
  The general appearance had 150 data items. Items 1 to 36 (100% agreement), items 37 to 88 (94.96% agreement), items 89 to 95 (98% agreement), items 96 to 106 (96% agreement), items 107 to 120 (86.76% agreement), items 121 to 134 (79.23% agreement), items 135 to 145 (89% agreement), and items 146 to 150 (88% agreement) were accepted in the first stage of Delphi, and therefore, all 150 general appearance items were accepted. Each item had a percentage of acceptance and the items whose acceptance percentage was close to each other are listed in a column.
I. Respiratory system
  The respiratory system had 140 data items. Items 1 to 55 (89.51% agreement), items 56 to 78 (98.23% agreement), items 79 to 100 (96.37% agreement), and items 101 to 125 (95% agreement) were accepted in the first stage of Delphi, and items 126 to 140 (79.80% agreement) were accepted in the second stage of Delphi and therefore, all 140 respiratory items were accepted. Each item had a percentage of acceptance and the items whose acceptance percentage was close to each other are listed in a column.

Due to the large amount of information, we had to provide, only Table 3, which is an example of a platform developed for cardiovascular nursing assessment.

**Table 3:** A sample of final nursing reporting MDS: the cardiovascular system assessment data elements

Data elements	Data values		
HR			
BP	Invasive		
	Non-invasive		
Iv line	Peripheral		Angio cat Scalp
	Central		Triple lumen Double lumen Port Cat down
Site of Iv line	Peripheral		Hand Leg Jugular
	Central		Subclavian Jugular Limb
Serum	Dose / Ml/hr. / Gtt/min		
Type of serum	Isotonic/ Hypertonic/ Hypotonic		
The patient needs blood products	No		
	Yes (if yes,)		FFP, Cryoprecipitate, Cryopoor Plasma (CPP), whole blood, platelet concentration, washed red blood cells, Low leukocyte red blood, Radiated red blood cells, Frozen red blood cells
Quality of pulse (power)	+, ++, +++, ++++		
Symptoms of dyspnea	PND (paroxysmal natural dyspnea), Orthopnea, dyspnea during exercise, dyspnea during rest,		
chest pain	yes		Onset, Site Radiation, Quality of pain, Pain aggravating factors, Pain Reduction Factors,
	no		
Capillary Refill time	Brisk (> 2 s), Sluggish (< 2)		
Time to start the pain			
CVP (central vein pressure)	Normal, abnormal		
Rhythm	normal		
	arrhythmia	Irregular	Atrium arrhythmia, Early Stimulation Syndromes, AV arrhythmia & blocks, Branch blocks, Ventricular arrhythmia,
		Regular	SA node arrhythmia
patient needs a pacemaker	yes		Internal, External
	no		
Set up of pacemaker	Mode, Output, Rate		
Does the patient need a monitor?	Yes, no		
Does the patient need an IABP?	Yes, no		setup
Edema	yes		Localize, general
	no		
Edema	yes		Pitting, Not pitting
	no		
CPCR	yes		onset start, duration, end time of CPCR, Time to announce resuscitation code, Drug are used, Type of rhythm, shock,
	no		
CPR successful	yes		post-CPR care
	no		
ventilate the patient during CPR	ETT, LMA, AMBO bag		

A sample of final clinical nursing reporting MDS: the Cardiovascular system assessment data elements

### Source of nursing diagnoses

In this study, we conducted a Delphi survey to determine the source for writing nursing diagnoses. For this purpose, the main sources for nursing diagnoses (NANDA, ANA, ICNP, CCC, and CINA) were selected and sent to the participants. One-hundred percent of the survey returned, and the NANDA classification was accepted with 98% approval. Nursing diagnoses were written based on NANDA and included in the final MDS of the nursing reporting system (Table 4).

**Table 4:** Source of nursing diagnoses

Nursing classification systems	Delphi survey rounds						Final Decision
	Round 1			Round 2			
	Agree N (%)	Dis agree N (%)	Unsure N (%)	Agree N (%)	Dis agree N (%)	Unsure N (%)	
NANDA	98	0	2				accepted
ANA	35.6	64.4	0				
ICNP	33.1	66.9					
CCC	45.2	54.8					
CINA	40.3	59.7					

Source of nursing diagnoses

### System evaluation

**Fig. 3:**
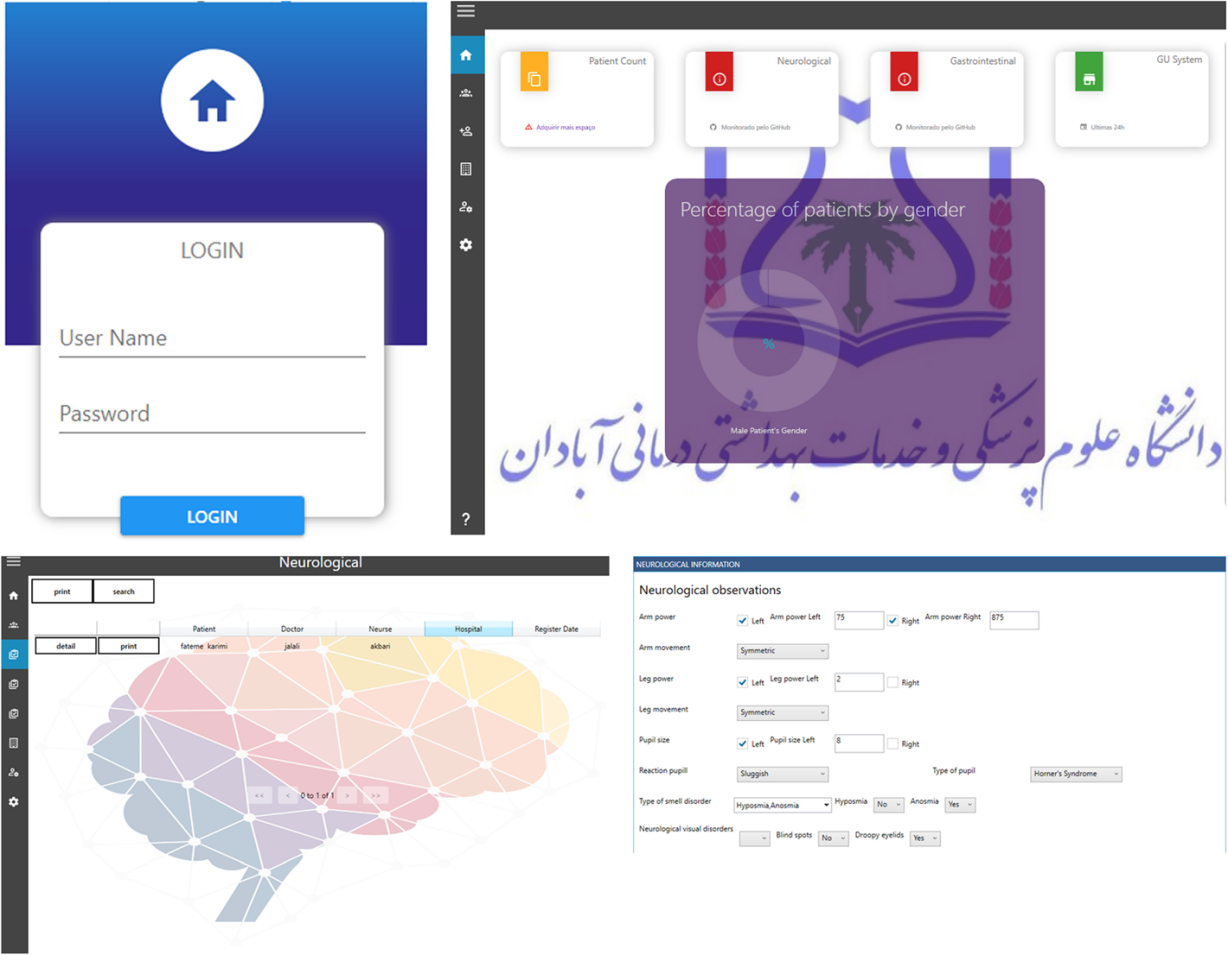
Clinical nursing documentation system home pages

After developing the ECNDS Fig. 3, it was experimentally used by the end-users (nurses), and a pilot study was performed on user satisfaction. This study was conducted on 150 clinical nurses. The majority of the participants were female (74.66%), and the average age was 36.4 (SD ± 6.4). About 45.33% of them were employed in the internal medicine-surgical wards, 35.33% worked in critical wards, 12.66% were employed in emergency wards, and 6.68% worked in other medical wards. The average work experience was 15.66 years (SD ± 4.5) (Table 5).

**Table 5:** Descriptive demographics of clinical nurses in the survey

Variables	Frequency	percentage
Gender		
female	112	74.66
male	38	23.34
ward		
ER	19	12.66
Critical care	53	35.33
Medical and surgical	68	45.33
Other	10	6.68
	mean	SD
Age	36.4	± 6.4
Work experience in clinical field (years)	15.66	± 4.5

Descriptive demographics of clinical nurses in the survey

**Fig. 4:**
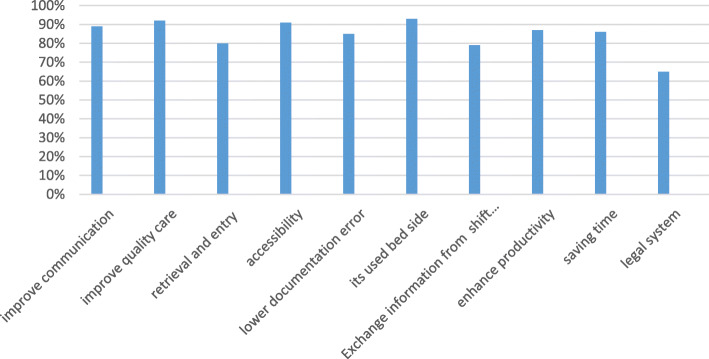
Positive impact of the ECNDS

**Fig. 5:**
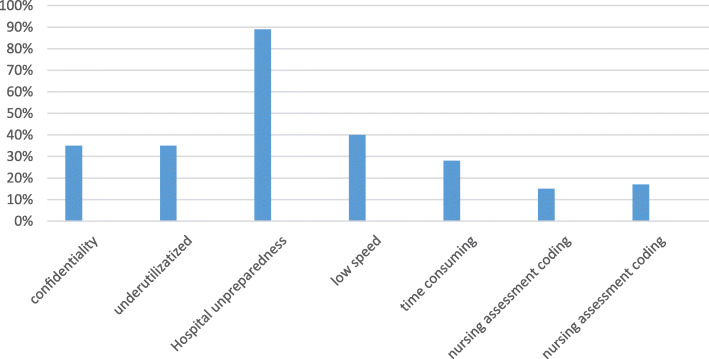
*Negative impact of the* ECNDS

The extracted data are classified into three categories. Figures 4 and 5 identify the positive and negative impacts of the developed ECNDS by nurses, respectively, after implementing the system at the Abadan hospitals.

The ECNDS can improve the communication between departments (89% of the nurses who responded to the question on communication agreed that the system functioned well and nurses were satisfied with it). This system can also improve the quality of patient care (92%). By using it, the retrieval and entry of patient information become simple and accurate (80%). In addition, the system is easy to access (91%), and can be used by nurses in different wards (89%). It also reduces documentation errors (85%); and legal issues (65%). The developed ECNDS is utilized used at bedside (93%), facilitates exchanging information from shift to shift (79%), and boosts the productivity (87%) (Fig. 4).

The second section of the survey is shown in Fig. 5 and describes the negative impacts of the ECNDS as follows: the loss of confidentiality (35% of the nurses answering the question on the confidentiality issue agreed that the ECNDS requires increased confidentiality); the ECNDS is dependent on utilities (35% of nurses agreed); the hospital is still using a paper system in some departments (89%); using the ECNDS is time consuming (28% agreed), and the system is works very slowly (60%). Moreover, 17% of the participants disagreed with the validity of coding the nursing diagnosis and 15% of them disagreed with the validity of the coding of the nursing assessment.

## Discussion

The field of information technology is developing and with its progress, it can help the advancement of other sciences such as nursing science. In Iran, nursing documentation is still conducted in a traditional and paper-based manner [8]. Electronic clinical nursing documentation needs the data to be stored according to a uniform and structured framework. This study aimed to design an electronic clinical documentation system for unified recording of nursing activities using standardized data elements which improve data quality, data interoperability, and decision-making, and pave the way for formulating global standards for nursing care. In this study, we conducted a systematic review study along with a two-round Delphi survey to prepare a formal and organized data structure and standard platform. This MDS could be applied to develop more patient-oriented, evidence-based, safe and high-quality nursing care. The developed MDS also helps to support decision-making. After the survey, the system was found that it prevents 65% of the legal issues, reduces 85% of documentation errors, and does not have the problems of the paper documentation process. After using the system, 92% of the nursing staff were satisfied with the increase in the quality of care, which is in line with a study in Oman [27]. In Iran, nursing documentation is a paper-based approach, and nurses spend about one to two hours completing nursing reports [28]. However, in this survey, 82% of the nurses stated that their time was saved by utilizing the system developed in this study. In our study, nurses’ satisfaction with filling nursing report documentation at the patients’ bedside, and exchanging information from one shift to another shift were 93 and 79%, respectively [12]. In other words, filling or documentation of the clinical nursing report at the patients’ bedside and exchanging patient information across shifts are two important features of a nursing report that were taken into account in our designed system. The ability of software to keep the information confidential is one of the most fundamental features of this tool. Due to the team’s support for its security, the system designed in our study received 65% satisfaction from nurses.

We discovered the benefits and drawbacks of EMR systems, from reviewed studies, e.g., equipment shortages and breakdowns, writing on paper and transferring to EMR [29], user-friendliness and interoperability, hardware and software problems, increased documentation load, lack of formal structure, inability to use at bedside, and other factors [30]. Therefore, in the system developed in our study, the following features have been considered to solve the major problems of EMR. Unlike other studies, our work has a formal and organized structure according to the nursing process, which has been shown to improve legislative compliance and completeness nursing documentation completeness [27, 30]. Our software indicated that with the advancement of EMR in nursing practice, many paper-based reporting problems such as; wasted time, disruption in design and clinical care the patients, medical errors, endangering patients’ safety, fading and illegibility of manual documentation, legal problems, and, other similar issues. Are resolved [8, 9]. The electronic system developed in this study allows the nurses to fill out the pre-designed standard platform at the patient’s bedside, eliminating the need to write on paper and transfer the information to the system. One of the time-consuming and non-user-friendly problems of EMR systems is recording reports on paper and transferring them to the system [29], which was solved in our software. On the other hand, this process is recorded at the patients’ bedside, and therefore, the patient’s information is not missed. Moreover, our software shows a graph of the progression of a patient’s clinical conditions such as the patient’s heart rate in different shifts. The mentioned features are among the strongest aspects of this system, which have been stated in a survey of clinical nurses. The simplicity and fluency of the designed platform as well as the existence of scientific and approved abbreviations in this platform are another advantage of this software noted in the survey by the nurses. Another key feature of this system is that it can be used in general and specialized clinical wards such as CCU. In the survey, nurses emphasized the software security factor in maintaining patient information and the support team of this system, which is a very important aspect in any software. Finally, we must mention that our designed software has the following features; (1) the existence of an official structure and approval by nursing professors; (2) it can be used in all hospitals and different wards;(3) it can be easily used in the bedside; (4) it has the ability to report nursing information from shift to shift; (5) after a survey of nurses working in the wards of Abadan hospitals, it was concluded that our system is user friendly, easy to learn and easy to used.

### Limitations

This study should have been used in more hospitals with more nurses; on the other hand, nurses were because of the heavy workload of nurses in clinical wards and their lack of spare time, it was difficult for them to participate in this study. In addition, nurses needed training use this system, took great effort. The infrastructure of some hospitals was not suitable for the components of this design and it was very challenging to build the necessary infrastructure.

## Conclusions

The primary purpose of the nursing documentation MDS was to scientifically reduce the amount of clinical nursing report data collected and documented by nurses during the patient care process, while also enhancing the enjoyment of nursing due to a reduction in documentation burden. Involvement of the system end-users in a meaningful way during the development process resulted in an easier conversion from paper-based to computerized documentation, higher approval from nurses who use the electronic nursing documentation system, and minimal complaints regarding its content in the practice setting.

## Data Availability

The datasets used and/or analyzed during the current study are available from the corresponding author on reasonable request.

## Abbreviations

EMR: electronic medical record
RN: register nurse
CSS: cascading style sheets
HTML: Hypertext Markup Language
NIS: Nursing information system
NANDA: North American Nursing Diagnosis Association International
NOC: Nursing Outcome Classification
NIC: Nursing Intervention Classification
ICNP: International Classification of Nursing Practice
ECNDS: electronic clinical nursing documentation system
MDS: minimal data sets
